# The association between the TERT rs2736100 AC genotype and reduced risk of upper tract urothelial carcinomas in a Han Chinese population

**DOI:** 10.18632/oncotarget.7777

**Published:** 2016-02-27

**Authors:** Xiaotian Yuan, Yan Meng, Ping Li, Nan Ge, Feng Kong, Liu Yang, Magnus Björkholm, Shengtian Zhao, Dawei Xu

**Affiliations:** ^1^ Department of Medicine, Division of Haematology and Centre for Molecular Medicine (CMM), Karolinska University Hospital Solna and Karolinska Institutet, Stockholm, Sweden; ^2^ Department of Urology and Central Research Laboratory, Shandong University Second Hospital, Jinan, China; ^3^ Nursing School, Shandong University, Jinan, China; ^4^ Karolinska Institutet-Shandong University Collaborative Laboratories for Cancer and Stem Cell Research, Jinan, China

**Keywords:** cancer risk, SNP, telomerase, TERT, urothelial cell carcinoma

## Abstract

Upper tract urothelial carcinomas (UTUCs) are originated from urothelium, and consist of renal pelvic carcinomas (RPCs) and ureter carcinomas (UCs). Most UTUCs have already become invasive when diagnosed and there is thus a need to identify high-risk populations for preventive intervention. Recent evidence has accumulated supporting common single nucleotide polymorphisms (SNPs) to be associated with increased risk of various malignancies. However, little is known about susceptibility loci in relation to UTUC development. We genotyped telomerase reverse transcriptase (TERT) rs2736100 variants, the SNP associated with a risk of multiple-types of cancer, in patients with UTUC (n = 212) and evaluated the relationship between the rs2736100 and UTUC risk by comparing to 289 healthy controls. Neither AA nor CC genotypes differed significantly between cases and controls, while the AC-carriers were associated with a reduced risk of UTUC compared to the homozygous AA (OR = 0.583; 95% CI: 0.388 − 0.875; *P* = 0.012) or AA + CC genotypes (0.613; 95% CI: 0.428 − 0.879; *P* = 0.010). Further analyses showed that the AC variant conferred a lower risk for early stage UTUCs or those with a wt TERT promoter. When UTUCs were sub-grouped into UCs and RPCs, the AC genotype still predicts a significantly lower risk for UC (*P* = 0.045, OR = 0.597, 95% CI: 0.370 − 0.963), while at a border line significance for RPC (*P* = 0.055, OR = 0.597, 95% CI: 0.324 − 0.976). Collectively, the rs2736100 AC variant predicts a reduced risk to develop UTUC.

## INTRODUCTION

Upper tract urothelial carcinomas (UTUCs) are originated from urothelium, and consist predominantly of renal pelvic carcinomas (RPCs) and ureter carcinomas (UTs) [1, 2]. Although not common, UTUC incidence has increased over the past two decades, and importantly, most of them have become invasive when diagnosed, mainly due to lack of early clinical symptoms and of useful screening and diagnostic tools [1–3]. Hence, the identification of a high-risk population and of reliable bio-markers for UTUCs is required to design better preventive intervention, to improve clinical diagnostics or management, and to contribute to personalized or precision medicine [1, 3]. Recent genome-wide association studies (GWAS) have provided strong evidence that common single nucleotide polymorphisms (SNPs) are associated with increased risk of human malignancies [4–7]. In UTUC etiology, genetic susceptibility and gene-environmental interaction or differences in the ability to counteract carcinogens has been suggested [1], however, little is known about the susceptibility loci to UTUC and so far only two UTUC-specific SNPs have been reported, including the variant allele SULT1A1*2 reducing sulfotransferase activity and the T-allele of rs9642880 on chromosome 8q24 [1, 8].

Telomerase reverse transcriptase (TERT) is the key catalytic component of telomerase responsible for lengthening telomeric DNA at the end of chromosomes [9–11]. The *TERT* gene is transcriptionally repressed and telomerase is silent in the majority of normal human somatic cells, whereas the TERT induction coupled with telomerase activation is required for malignant transformation and occurs widely in human cancer including UTUCs [9, 10, 12]. It has been well established that the aberrant TERT expression confers cancer cells not only unlimited proliferative potential by stabilizing telomere sizes, but also aggressive phenotypes via its telomere lengthening-independent mechanisms [9, 10, 13–19]. Given the fundamental role of TERT in oncogenesis, much attention has been paid to the association between single nucleotide variants or SNPs of the *TERT* gene and cancer susceptibility, among which rs2736100 (located in intron 2) is most studied and its variants associated with risk of multiple-types of cancer, as documented by many published reports [4, 5, 7, 20–35]. However, it is currently unclear whether there exist any links between rs2736100 variants and UTUC risk.

In addition to germline TERT variants contributing to cancer risk as described above, more recently, the hotspot TERT promoter mutations named C228T and C250T were identified as a key genetic event to activate telomerase in different types of cancer [36–38]. We found that approximately 50% of RPCs and 20% of UCs carried TERT promoter mutations [2, 3, 39]. Because the cancer-risk alleles of the TERT SNPs may contribute to cancer susceptibility by their regulatory effect on TERT expression and telomerase activity [26, 40], it is worth assessing a relationship between TERT risk-alleles and TERT promoter mutations, which likely provides insights into cooperative roles of germline variants and somatic mutations of the *TERT* gene in oncogenesis.

In the present study, we sought to address the above issues by genotyping rs2736100 SNPs in UTUC patients and healthy adult controls.

## RESULTS

### Patient characteristics

A total of 212 patients with UTUC were genotyped for rs2736100 variants and they included 92 RPCs and 120 UCs. Clinical-pathological characteristics of these patients, including sex, age at diagnosis, stage, grade and metastases, are summarized in Table 1.

**Table 1:** Clinical characteristics of patients with upper tract urothelial carcinoma (UTUC)

informative cases (n = )	RPC*	UC*	Total
	92	120	212
***Age at diagnosis (n = 212)***			
Mean ± SD	63 ± 11	66 ± 11	64 ± 11
Median (range) years	64 (36 - 85)	67 (32 - 87)	66 (32 - 87)
***Gender (n = 212)***			
Female	37	45	82
Male	55	75	130
***Metastases or capsular invasion (n = 189)***			
Yes	6	11	17
No	77	95	172
***Stages (n = 189)***			
Pa + I	16	24	40
PII + III + IV	67	82	149
***grades (n = 189)***			
G1	13	12	25
G2	9	13	22
G3	61	81	142

* RPC, Renal pelvic carcinoma; UC, Ureter carcinoma.

### rs2736100 SNPs and UTUC risk

Table 2 lists the genotype distributions of TERT rs2736100 A>C in both adult healthy controls and UTUC patients. The genotype frequencies were 29.8%, 49.8% and 20.4% for AA, AC and CC, respectively, in healthy controls, while 39.2%, 38.2% and 22.6% for AA, AC and CC, respectively, in UTUC patients (Table 2). The prevalence of the rs2736100 heterozygous AC genotype was significantly lower in UTUC patients than in healthy controls, indicating a reduced risk for UTUC [Odds ratio (OR) = 0.583; 95% Confidence interval (CI): 0.388 - 0.875; *P* = 0.012] (Table 2) when the homozygous AA variant was used as a reference. We then combined the AA and CC genotypes together and compared them with the AC variant, and a significant difference was also obtained (0.613; 95% CI: 0.428 - 0.879; *P* = 0.010) (Table 2). When UCs and RPCs were analyzed separately, there were no significant differences in the allele distribution between RPCs and UCs (*P* = 0.257) (Table 2 and data not shown); the AC genotype remained significantly associated with a lower risk for UCs (*P* = 0.031, OR = 0.57, 95% CI: 0.35 - 0.93), while boarder-line significant for RPCs, compared to the AA variant (OR = 0.562, 95% CI: 0.324 – 0.976, *P* = 0.055) (Table 2). When combining the AA and CC variants together as a reference, the AC allele was associated with a significantly decreased risk of RPCs (OR = 0.537, 95% CI: 0.330 – 0.874, *P* = 0.016), however, despite a lower frequency of the A/C variant and a lower OR value in UCs, the difference did not reach a statistically significant level (OR = 0.695, 95% CI: 0.452 – 1.069, *P* = 0.121) (Table 2).

**Table 2:** TERT rs2736100 genotypes in healthy controls and patients with upper tract urothelial carcinoma (UTUC)

Genotype	Control	RPC*			UC*			Total UTUC		
	N (%)	N (%)	Odds ratio (95% CI*)	P	N (%)	Odds ratio (95% CI*)	P	N (%)	Odds ratio (95% CI*)	P
***rs2736100 (N/%)***	289	92			120			212		
AA	86 (29.8)	34 (37.0)	1.0 (ref.)		49 (40.8)	1.0 (ref.)		83 (39.2)	1.0 (ref.)	
AC	144 (49.8)	32 (34.8)	0.562 (0.324 - 0.976)	0.055	49 (40.8)	0.597 (0.370 - 0.963)	**0.045**	81 (38.2)	0.583 (0.388 - 0.875)	**0.012**
CC	59 (20.4)	26 (28.2)	1.115 (0.606 - 2.049)	0.846	22 (18.4)	0.714 (0.396 - 1.288)	0.330	48 (22.6)	0.878 (0.542 - 1.423)	0.685
AA + CC	145 (50)	60 (65)	1.0 (ref.*)		71 (59.2)	1.0 (ref.)		133(62.1)	1.0 (ref.)	
AC	144 (49.8)	32 (35)	0.537 (0.330 - 0.874)	**0.016**	49 (40.8)	0.695 (0.452 - 1.069)	0.121	81 (37.9)	0.613 (0.428 - 0.879)	**0.010**

* RPC, renal pelvic carcinoma; UC, Ureter carcinoma; C, confident interval; Ref., reference.

### The rs2736100 AC genotype association with early stages of UTUCs

The rs2736100 C allele has been shown to be associated with progressive disease and/or poor prognosis in certain malignancies [24, 31]. To see if this was the case in UTUCs, we analysed the rs2736100 variants according to disease stage and grade, and observed a significantly negative association between the heterozygous rs2736100 AC genotype and early stages (pTa + T1) of UTUCs (OR = 0.358, 95% CI: 0.167 - 0.769, *P* = 0.012) (Table 3). No significant difference in the rs2736100 allele frequency was found in relation to histological grade of UTUCs, as shown in Table 3.

**Table 3:** Association of TERT rs2736100 variants with pathological variables and TERT promoter mutations in patients with upper tract urothelial carcinoma (UTUC)

*Genotype*	Cases	Healthy controls	Odds ratio (95% CI)	P
***Stages pTa - I vs controls***				
AA	20 (48.8%)	86 (29.8%)	1.0 (Ref.)	
AC	12 (29.2%)	144 (49.8%)	0.358 (0.167 - 0.769)	0.012
CC	9 (22.0%)	59 (20.4%)	0.656 (0.279 - 1.540)	0.455
***Stages pII + III + IV vs controls***				
AA	50 (33.8%)	86 (29.8%)	1.0 (Ref.)	
AC	64 (43.2%)	144 (49.8%)	0.764 (0.484 - 1.206)	0.229
CC	34 (23.0%)	59 (20.4%)	0.991 (0.573 - 1.713)	0.914
***Grade G1 vs controls***				
AA	10 (41.7%)	86 (29.8%)	1.0 (Ref.)	
AC	8 (33.3%)	144 (49.8%)	0.478 (0.182 - 1.257)	0.203
CC	6 (25.0%)	59 (20.4%)	0.875 (0.301 - 2.537	0.983
***Grade G2 + G3 vs controls***				
AA	60 (36.4%)	86 (29.8%)	1.0 (Ref.)	
AC	68 (41.2%)	144 (49.8%)	0.675 (0.437 - 1.049)	0.101
CC	37 (22.4%)	59 (20.4%)	0.899 (0.531 - 1.522)	0.793
***wt TERT promoter vs controls***				
AA	50 (37.5%)	86 (29.8%)	1.0 (Ref.)	
AC	50 (37.5%)	144 (49.8%)	0.597 (0.372 - 0.960)	0.044
CC	33 (25.0%)	59 (20.4%)	0.962 (0.555 - 1.668)	0.988
***mt TERT promoter vs controls***				
AA	20 (35.7%)	86 (29.8%)	1.0 (Ref.)	
AC	26 (46.4%)	144 (49.8%)	0.776 (0.409 - 1.474)	0.543
CC	10 (17.9%)	59 (20.4%)	0.729 (0.318 - 1.668)	0.586

CI: confidence interval

### TERT promoter mutations and association with rs2736100 in UTUCs

Because a higher frequency of the TERT promoter mutation was previously observed in UTUCs and this genetic event plays a key part in activating telomerase [2, 3, 39], we further sought to determine whether there is a link between this mutation and rs2736100 variants. The TERT promoter was sequenced in UTUC tumors and 56 of 189 evaluable patients (30%) carried a C228T or C250T mutation. We divided UTUC patients into two groups: UTUCs with a wt and mutant TERT promoter, respectively and observed that the AC allele was negatively associated with wt promoter-carrying UTUCs (OR = 0.597 (95% CI: 0.372 – 0.960, P = 0.044), whereas there was no association between the same variant and those with a mutant TERT promoter (Table 3).

## DISCUSSION

In the present study, we analyzed TERT rs2736100 SNPs in UTUC patients and age/sex- matched healthy individuals, and our findings demonstrated a significant association between TERT SNPs and risk of UTUC: The rs2736100 AC genotype predicts a reduced UTUC risk. Moreover, this variant was also negatively associated with an early disease stage and wt TERT promoter. To our knowledge, this is the first report on the relation between TERT SNPs and UTUC susceptibility.

The relationship between the rs2736100 SNP and cancer susceptibility has been extensively explored [4, 5, 20–32, 35, 40, 41]. We and others previously analyzed the rs2736100 association with lung cancer risk, and observed a significantly elevated risk in C variant-carriers [20]. Recently, rs2736100-C was further identified to be more intimately associated with female, non-smoking, EGFR-mutation-positive lung adenocarcinoma [40]. In addition, the rs2736100-C has also been shown to be a risk allele for malignant glioma, colorectal carcinoma, cervical, pancreatic, bladder, and ovarian cancer, acute myeloid and lymphocytic leukemia, and others. [6, 42–44]. Intriguingly, however, the risk allele seems to differ in different types of cancer. The rs2736100-A allele was significantly associated with a higher risk of testicular cancer [41]. We found that the rs2736100 AC allele-carriers exhibited a lower risk of UTUC than those with AA and CC variants, while a previous analysis showed the AC genotype was a risk allele for bladder cancer in Chinese population [27]. These data indicate a complicated relationship between the rs2736100 SNP and cancer susceptibility.

Urothelial bladder carcinomas (UBCs), as UTUCs, are also derived from the ureothelium [45–47]. Both UTUCs and UBCs share common oncogenic alterations. For instance, TERT promoter and *fibroblast growth factor receptor 3* (*FGFR3*) gene mutations are not only the most frequent genetic events in UBCs [45], but also widespread in UTUCs including both RPC and UC [46]. In particular, 60 to 80% of non-muscle-invasive UBCs harbor *FGFR3* mutations and the mutations are similarly associated with low-stage or non-invasive UTUCs [45, 46]. FGFR3 is a tyrosine kinase receptor that mediates the effects of fibroblast growth factors (FGFs) and stimulates the RAS-mitogen-activated protein kinase (MAPK) and phosphatidylinositide-3 kinase–AKT pathway. Intriguingly, rs2736100 risk alleles for UTUCs and UBCs are completely different. The C allele was observed to be associated with an increased risk for UBCs in the Han Chinese population [27], opposite to the present finding in UTUCs. Our results apparently add further layers of complexity and raise more questions regarding the role of rs2736100 risk-alleles in oncogenesis.

TERT plays a key role in oncogenesis via both telomere lengthening-dependent and independent activities, and it is thus not surprising to observe an intimate association between its SNPs and cancer risk. However, it remains unclear how these TERT variants contribute to cancer susceptibility. In a few of studies, the effect of rs2736100 alleles on TERT expression or telomerase activity was analyzed, but the results were discrepant. Wei et al observed that the luciferase reporter driven by C allele-containing sequences exhibited a higher activity than that by A allele-carrying fragments in one lung cancer cell line; they also showed significantly higher TERT mRNA levels coupled with longer telomere in lung cancer patients harboring CC genotypes than those with AA or AC variant [40]. In contrast, however, in patients with acute lymphoblastic leukemia, leukemic cells derived from CC- and AC-allele-carriers expressed lower levels of telomerase activity and had shorter telomere compared to those with the AA genotype [26]. Moreover, there did not exist DNA hypersensitivity sites and histone methylation at rs2736100 [30], which does not support a direct regulatory effect of this SNP on TERT expression. These results collectively suggest that the cancer risk resulting from TERT SNPs may not be simply attributable to their roles in controlling telomerase activity or telomere length.

The hotspot TERT promoter mutations named C228T and C250T were recently identified as a key genetic event to activate telomerase in different types of cancer including urothelial cell carcinomas [2, 3, 38, 39]]. The mutations create extra ETS binding motifs in the proximal TERT promoter and thereby activate the TERT gene transcription. If the rs2736100 risk allele exerts its effect by up-regulating TERT or telomerase expression, we may expect that the risk allele is more frequent or the protective allele is fewer in wt TERT promoter-carrying tumors. In the present cohort of UTUC patients, 30% of them harbored C228T or C250T mutations, and indeed, we observed that the AC variant, a protective allele, was significantly associated with the reduced risk for wt TERT promoter-carrying UTUCs. Moreover, such an association was found between the rs2736100 AC genotype and Ta and T1 stages of UTUCs, while the TERT promoter mutation was less frequent in UTUCs at these stages.

In summary, the present finding reveals that the rs2736100 AC genotype predicts a reduced UTUC risk, especially in UTUCs with wt TERT promoter at early stages. These results are unexpected and opposite to those describing the AC variants as risk-alleles in other cancer types. Likely, UTUCs have unique genetic risk factors and etiological pathways that should be addressed in future studies.

## MATERIALS AND METHODS

### Study populations

Two hundred and twelve newly diagnosed, histologically confirmed sporadic UTUC patients were recruited from the Shandong University Qilu and Second Hospitals. Adult healthy individuals served as controls were age- (±9 years) and sex-matched. The ethnic background of both patients and controls was Han Chinese. Tumor specimens and/or blood samples were obtained from the participants with informed content. The study was approved by the Shandong University Second Hospital Ethical Committee.

### DNA extraction and genotyping

Genomic DNA was extracted using QIAGEN DNA extraction kits. The rs2736100 (AC) genotyping was performed using pre-designed TaqMan SNP genotyping assay kits on an ABI PRISM 7900 HT Sequence Detection System (Applied Biosystems). Each assay included both positive and negative controls and was run with the following condition: 95°C for 10 min, followed by 40 cycles of 92°C for 15 s and 60°C for 1 min.

### Sanger sequencing of the proximal TERT promoter region

DNA extracted from patient tumor specimens was analyzed for the *TERT* gene promoter mutation by Sanger sequencing using the primer pairs as previously des cribed [38, 48]: 5b 2-CACCCGTCCTGCCCCTTCACCTT-3b 2 (forward) and 5b 2-GGCTTCCCACGTGCGCAGCAGGA-3b 2 (reverse). All the mutations were verified by sequencing from both directions.

### Statistical analyses

Age and sex were compared between patients and healthy controls using the Mann–Whitney *U* and Chi-square (χ^2^) tests, respectively and there is no difference. The evaluation of distribution differences of selected variables and allele of the *TERT* polymorphisms between patients and healthy controls were done using χ^2^ test. Hardy–Weinberg equilibrium of the genotype distribution among the controls was tested by a goodness-of-fit χ^2^ test. Unconditional univariate and multivariate logistic regression analyses were used to estimate ORs for risk of UTUC and their 95% CIs. All the tests were computed using SigmaStat3.1® software (Systat Software, Inc., Richmond, CA). *P* values <0.05 were considered as statistically significant.
